# Transforming Medical Imaging: The Role of Artificial Intelligence Integration in PACS for Enhanced Diagnostic Accuracy and Workflow Efficiency

**DOI:** 10.2174/0115734056370620250403030638

**Published:** 2025-04-22

**Authors:** Alberto I. Pérez-Sanpablo, Jimena Quinzaños-Fresnedo, Josefina Gutiérrez-Martínez, Irma G. Lozano-Rodríguez, Ernesto Roldan-Valadez

**Affiliations:** 1 Research Division, Human Motion Analysis Laboratory and Rehabilitation Engineering Department, National Institute of Rehabilitation Luis Guillermo Ibarra Ibarra, 14389, Mexico City, Mexico; 2 Neurologic Rehabilitation Division, National Institute of Rehabilitation Luis Guillermo Ibarra Ibarra, 14389, Mexico City, Mexico; 3 Medical Engineering Research Division, National Rehabilitation Institute “Luis Guillermo Ibarra Ibarra”, 14389, Mexico City, Mexico; 4 Division of Research, National Rehabilitation Institute 'Luis Guillermo Ibarra Ibarra', 14389, Mexico City, Mexico; 5 Department of Radiology, I.M. Sechenov First Moscow State Medical University (Sechenov University), 119992, Moscow, Russia

**Keywords:** Artificial intelligence, PACS, Medical imaging, Diagnostic accuracy, Machine learning, Deep learning, Cloud computing, Natural language processing, Interoperability, Healthcare systems

## Abstract

**Introduction:**

To examine the integration of artificial intelligence (AI) into Picture Archiving and Communication Systems (PACS) and assess its impact on medical imaging, diagnostic workflows, and patient outcomes. This review explores the technological evolution, key advancements, and challenges associated with AI-enhanced PACS in healthcare settings.

**Methods:**

A comprehensive literature search was conducted in PubMed, Scopus, and Web of Science databases, covering articles from January 2000 to October 2024. Search terms included “artificial intelligence,” “machine learning,” “deep learning,” and “PACS,” combined with keywords related to diagnostic accuracy and workflow optimization. Articles were selected based on predefined inclusion and exclusion criteria, focusing on peer-reviewed studies that discussed AI applications in PACS, innovations in medical imaging, and workflow improvements. A total of 183 studies met the inclusion criteria, comprising original research, systematic reviews, and meta-analyses.

**Results:**

AI integration in PACS has significantly enhanced diagnostic accuracy, achieving improvements of up to 93.2% in some imaging modalities, such as early tumor detection and anomaly identification. Workflow efficiency has been transformed, with diagnostic times reduced by up to 90% for critical conditions like intracranial hemorrhages. Convolutional neural networks (CNNs) have demonstrated exceptional performance in image segmentation, achieving up to 94% accuracy, and in motion artifact correction, further enhancing diagnostic precision. Natural language processing (NLP) tools have expedited radiology workflows, reducing reporting times by 30–50% and improving consistency in report generation. Cloud-based solutions have also improved accessibility, enabling real-time collaboration and remote diagnostics. However, challenges in data privacy, regulatory compliance, and interoperability persist, emphasizing the need for standardized frameworks and robust security protocols.

**Conclusion:**

The integration of AI into PACS represents a pivotal transformation in medical imaging, offering improved diagnostic workflows and potential for personalized patient care. Addressing existing challenges and enhancing interoperability will be essential for maximizing the benefits of AI-powered PACS in healthcare.

## 1 INTRODUCTION

### 1.1 Overview of PACS and Its Evolution

The integration of artificial intelligence (AI) into Picture Archiving and Communication Systems (PACS) represents a paradigm shift in medical imaging, fundamentally altering the way diagnostic workflows are conducted. As healthcare increasingly adopts digital transformation, the role of AI has expanded from simple automation to enabling precision diagnostics and personalized care. PACS systems, traditionally designed for image storage and management, are now leveraging AI technologies such as deep learning (DL) and natural language processing (NLP) to address critical challenges, including rising diagnostic workloads, variability in radiological interpretations, and the need for faster decision-making. By automating complex tasks like image segmentation, anomaly detection, and predictive analytics, AI integration in PACS offers a scalable solution to improve efficiency and accuracy across healthcare systems [1, 2]. The primary aim of PACS was to create a centralized digital repository for various imaging modalities such as X-ray, CT, MRI, and ultrasound, thereby streamlining radiological workflows and enhancing diagnostic efficiency [2]. This transformation is particularly significant in the context of the global rise in imaging demands. For instance, radiologists face an average increase in workload of 6% per year, underscoring the urgency for innovative solutions to maintain diagnostic accuracy without delays. AI-powered PACS systems not only streamline these processes but also enable broader access to high-quality imaging services through advancements in cloud computing and interoperability frameworks. This review aims to explore these transformative capabilities, while addressing the challenges and opportunities that AI integration brings to modern radiology [3]. Fig. (**1**) illustrates a timeline of key milestones in the evolution of artificial intelligence (AI) and machine learning (ML) in medical imaging. The timeline begins in the 1980s with early academic research into machine learning applications for medical devices. The 1990s mark the introduction of the first graphics processing units (GPUs), which significantly enhanced computational power, enabling the rapid processing of complex medical image data. This decade also saw the first Food and Drug Administration (FDA) approval of an AI-based device for mammography, highlighting a crucial regulatory step in validating AI applications for clinical use. Moving into the 2010s, the figure highlights transformative breakthroughs in deep learning, exemplified by advancements in the ImageNet challenge, and the integration of cloud computing, which facilitated scalable AI training and data analysis. The timeline progresses into the 2020s, showcasing the emergence of federated learning-an innovative approach that allows AI models to be trained across multiple decentralized data sources while preserving patient privacy. Additionally, the use of AI for automated reporting in medical imaging has become more prevalent, supported by enhanced computational capabilities. This chronological overview captures the progressive integration of AI into medical imaging, underscoring its transformative impact on diagnostic workflows and the capabilities of PACS.

#### 1.1.1 Early Development and Adoption

The early stages of PACS development focused on providing a centralized storage system for digital images across multiple imaging modalities, significantly improving access to radiological data within medical facilities [2]. By eliminating the need for physical film storage, PACS facilitated faster image retrieval and enhanced clinical decision-making, allowing healthcare professionals to collaborate more effectively [3].

#### 1.1.2 Integration with Radiology and Hospital Systems

As PACS evolved, it became integrated with Radiology Information Systems (RIS) and Hospital Information Systems (HIS), significantly enhancing departmental workflows and minimizing data discrepancies [4, 5]. Standardization efforts, such as the adoption of Digital Imaging and Communications in Medicine (DICOM) and Health Level Seven (HL7) protocols, have been critical in achieving interoperability between different systems, ensuring consistent communication of imaging data across various devices and platforms [2, 6]. These integrations have streamlined diagnostic processes, reduced the likelihood of errors, and improved patient data management efficiency.

Recent data underscores the widespread adoption of Picture Archiving and Communication Systems (PACS) and the increasing role of artificial intelligence (AI) in radiology. In 2022, the demand for PACS in hospitals accounted for 15.9% of the market share, with expectations to surge at a compound annual growth rate (CAGR) of 17% between 2023 and 2033, driven by the growing adoption of advanced technologies to safely store patient data and improve treatment outcomes [7]. The global AI in medical imaging market was valued at USD 1.01 billion in 2023 and is projected to grow at a CAGR of 34.8% from 2024 to 2030, reflecting the rising demand for AI-based solutions to enhance diagnostic accuracy and reduce radiologists' workload [8].

These trends highlight the critical role of AI in addressing the increasing demand for imaging services and improving diagnostic accuracy and efficiency, providing a strong foundation for exploring its integration into PACS.

#### 1.1.3 Recent Advances and Trends

Recent developments in PACS have been driven by the integration of AI and ML technologies, which aim to enhance diagnostic capabilities [9, 10]. AI algorithms now assist in image analysis, automate routine tasks, and provide clinical decision support, improving both diagnostic accuracy and efficiency. The shift towards cloud-based PACS solutions has further enhanced accessibility, enabling remote viewing and collaboration-particularly valuable during the COVID-19 pandemic [6, 11]. Cloud-based systems reduce the need for extensive on-site infrastructure, offer scalable storage, and facilitate real-time data updates [5, 6].

The transformative potential of AI is evident across multiple domains, providing a context for its integration into Picture Archiving and Communication Systems (PACS). For instance, AI-driven advancements have revolutionized immersive educational tools, such as gamified augmented reality and virtual reality platforms in agricultural education, which enhance engagement and retention rates through interactive learning environments [12]. Similarly, AI-powered customer service models have optimized user experiences by employing natural language processing and predictive analytics to address customer inquiries more effectively [13].

In finance, studies evaluating AI’s influence on market values underscore the distinction between authentic growth driven by data insights and speculative trends fueled by algori-thmic trading, shedding light on AI’s potential for creating sustain-able economic models. These findings are pertinent when considering how AI can differentiate meaningful diagno-stic improvements from superficial automation in PACS [14].

In healthcare, AI has demonstrated its value in cancer diagnosis with architectures that integrate graduated levels of trust, ensuring clinical reliability while minimizing false positives and negatives [15]. Recent advancements in diagnostic models, such as a multivariable approach for identifying unusual infections in hospitalized patients, highlight AI’s capacity for integrating diverse clinical data [16]. Additionally, foundational AI models like Med-SAM1 and Med-SAM2 have showcased remarkable performance in segmenting complex 3D MRI structures, including left atrial segmentation in LGE MRI, which parallels the imaging challenges tackled by AI-powered PACS [17].

These advancements across domains demonstrate the versatility and transformative potential of AI, providing a strong foundation for exploring its integration into PACS to address diagnostic, workflow, and interoperability challenges.

### 1.2 The Rise of AI in Medical Imaging

Artificial Intelligence (AI) has emerged as a key innovation in medical imaging, driving the development of advanced diagnostic tools that enhance patient care. The integration of AI with PACS has led to significant improvements in image interpretation, faster diagnostic workflows, and reduced workloads for radiologists [10, 18]. AI technologies, including deep learning and natural language processing (NLP), have expanded the capabilities of PACS by enabling real-time data analysis and predictive analytics [19].

To illustrate the sequential development and integration of AI and machine learning (ML) technologies within medical imaging, we present a comprehensive overview of the AI/ML lifecycle. The lifecycle highlights critical stages from the initial design and development of AI models to their real-world clinical implementation. This framework helps to contextualize the systematic process involved in creating robust, reliable, and ethically sound AI applications, bridging the gap between innovative research and clinical practice. The following Fig. (**2**) summarizes these stages, offering a clear visual representation of the steps involved in advancing AI-powered diagnostic tools in medical imaging.

#### 1.2.1 AI Advancements and Clinical Applications

AI algorithms are widely used for analyzing medical images, detecting patterns, and identifying anomalies that may be difficult for radiologists to discern. These capabilities are crucial for early diagnosis and treatment planning [19]. For instance, AI-enhanced MRI applications have shown improvements in scanning speed, image resolution, and reduced radiation exposure, which are essential for specialties like neurology, orthopedics, and oncology [20, 21]. Additionally, deep learning techniques have proven effective in reducing noise and correcting motion artifacts in MRI, further enhancing image quality [6]. In brain imaging, AI algorithms have demonstrated high accuracy in detecting early-stage tumors, underscoring their transformative impact [22].

#### 1.2.2 Regulatory Approvals and Market Growth

The growing reliance on AI in medical imaging is reflected in the increasing number of regulatory approvals. As of 2023, the U.S. Food and Drug Administration (FDA) had cleared over 700 AI-based medical imaging algorithms, with approximately 76% focused on radiology applications [23]. This trend highlights the expanding role of AI in improving diagnostic precision and efficiency. The global market for AI in medical imaging is projected to grow significantly, with estimates reaching $11.76 billion by 2033 [24].

#### 1.2.3 Technological Integration and Future Perspectives

The integration of AI into medical imaging extends beyond image quality enhancement to include automation of complex tasks such as image segmentation and classification. At the 2023 Radiological Society of North America (RSNA) conference, AI was showcased for its role in automating diagnostic processes and providing robust clinical decision support [25]. AI tools now streamline intricate tasks like tracking tumor size, aiding radiologists in effective case management [23]. Table **1** summarizes the evolution of PACS and the key milestones in AI integration over the past five decades.

### 1.3 Justification of the Review's Importance

The integration of AI into PACS marks a pivotal advancement in medical imaging, with profound implications for diagnostic accuracy, workflow optimization, and patient care. This review is both timely and crucial for guiding healthcare professionals and researchers in navigating the evolving landscape of AI-powered PACS. By examining current trends and projecting future directions, this article aims to be an essential resource for leveraging AI to enhance PACS capabilities and address ongoing challenges in the field. The following bullet points outline the key contributions of this review:

- Highlight the transformative role of AI integration in PACS to improve diagnostic accuracy and workflow efficiency.
- Present a comprehensive review of the technological advancements in AI, including deep learning and natural language processing applications in medical imaging.
- Address critical challenges such as interoperability, data privacy, and regulatory compliance for AI-powered PACS adoption.
- Identify emerging trends and future directions for AI in radiology, with a focus on improving patient outcomes and clinical workflows.
- Propose practical recommendations to enhance the integration of AI into PACS systems based on existing studies and technological innovations.

### 1.4 Aims and Reader Benefits

This article is a narrative review that synthesizes advancements, challenges, and future directions in the integration of artificial intelligence (AI) into Picture Archiving and Communication Systems (PACS). Unlike a systematic review, it provides a focused exploration of key developments and clinical applications without exhaustive coverage of all eligible studies. By highlighting the transformative effects of AI on diagnostic workflows and accuracy, this article addresses key challenges and proposes innovative solutions for seamless AI integration. Readers will gain insights into how AI-enhanced PACS can streamline clinical processes, reduce diagnostic errors, and improve patient outcomes, making this review a valuable resource for clinicians, researchers, and industry stakeholders.

### 1.5 Proposed Section Plan

The next sentences show a concise overview of the manuscript’s organization, outlining the content of each major section.

**Section 2**: Discusses the methods used for the literature search, data extraction, and synthesis, providing a framework for analyzing AI integration into PACS.

**Section 3**: Reviews the technological foundations and key applications of AI in PACS, including diagnostic enhancements, workflow optimization, and emerging innovations.

**Section 4**: Examines the Challenges and Barriers to AI-PACS Integration, focusing on data privacy, regulatory compliance, and interoperability issues.

**Section 5**: Explores the preparedness of current PACS infrastructures for AI integration and proposes strategies to overcome existing limitations.

**Section 6**: Highlights clinical applications and case studies demonstrating the effectiveness of AI-powered PACS in various medical specialties.

**Section 7**: Discusses ethical considerations and patient-centered perspectives in the adoption of AI technologies in PACS.

**Section 8**: Summarizes future directions and trends for AI integration in PACS, emphasizing the need for innovation and collaboration in radiology.

## 2 METHODS: COMPREHENSIVE APPROACH TO ANALYZING AI IN PACS

This narrative review was designed to provide an in-depth analysis of the integration of artificial intelligence (AI) into Picture Archiving and Communication Systems (PACS). The review follows a structured approach for literature search, data selection, extraction, and synthesis to ensure comprehensive coverage of the most recent developments, key challenges, and emerging trends in the field.

### 2.1 Literature Search Strategy

A comprehensive literature search was conducted using three major scientific databases: PubMed, Scopus, and Web of Science. The search covered the period from January 2000 to October 2024, ensuring the inclusion of the most recent and relevant literature. The following Boolean search strategy was applied:

(“artificial intelligence” OR “machine learning” OR “deep learning”) AND (“PACS” OR “Picture Archiving and Communication Systems”) AND (“medical imaging” OR “diagnostic accuracy” OR “workflow efficiency”).

Filters Applied:

- *Language*: English-only articles were included to ensure clarity and accessibility of the content.
- *Publication Type*: Peer-reviewed studies, systematic reviews, meta-analyses, and original research articles were included, while editorials, commentaries, and opinion pieces were excluded.
- *Relevance*: Articles were selected based on their focus on AI applications in PACS, innovations in medical imaging, and workflow enhancements. Conference abstracts without full-text availability were excluded.

The literature search focused on identifying significant advancements and challenges in AI integration into PACS. Articles were selected based on their relevance, impact, and contribution to the field, as determined by expert evaluation of the topic.

### 2.2 Inclusion and Exclusion Criteria

To ensure the relevance and quality of the included studies, predefined inclusion and exclusion criteria were applied:

Inclusion Criteria:

- Articles published in English.
- Peer-reviewed studies focusing on AI applications in medical imaging, particularly within PACS.
- Research that discusses the impact of AI on PACS workflows, diagnostic accuracy, patient outcomes, and data management.
- Studies involving innovations in image analysis, segmentation, classification, and predictive analytics.

Exclusion Criteria:

- Non-English articles.
- Editorials, commentaries, and opinion pieces lacking substantial data.
- Conference abstracts without full-text availability.
- Studies unrelated to the integration of AI into PACS or focusing on non-medical applications of AI.

### 2.3 Data Extraction

Data extraction was conducted using a standardized data collection form. The extracted information included the following key aspects:

- **Study characteristics**: Author(s), year of publication, study type (*e.g.*, original research, systematic review).
- **AI techniques and models**: Type of AI algorithm (*e.g.*, convolutional neural networks, natural language processing, generative adversarial networks).
- **Applications in PACS**: Specific AI applications (*e.g.*, image segmentation, anomaly detection, automation of workflows).
- **Outcomes**: Reported effects on diagnostic accuracy, workflow efficiency, and patient outcomes.
- **Challenges and limitations**: Identified barriers to AI integration, such as data privacy concerns, regulatory issues, and technological limitations.

Two independent reviewers performed the data extraction to minimize bias and ensure the accuracy of the collected information. Discrepancies were resolved through discussion or consultation with a third reviewer.

### 2.4 Data Synthesis

The extracted data were synthesized qualitatively to provide a comprehensive overview of AI integration into PACS. The synthesis focused on identifying common themes, key advancements, and challenges in the field. The findings were categorized into major thematic areas:

- **AI-driven image processing and analysis**: Innovations in image segmentation, enhancement, and classification.
- **Workflow optimization and automation**: AI applications in streamlining diagnostic processes, including worklist prioritization and automated reporting.
- **Regulatory and ethical considerations**: Challenges related to data privacy, algorithmic bias, and regulatory compliance.
- **Future trends and emerging technologies**: Potential advancements in AI-powered PACS, such as the integration of augmented reality (AR), virtual reality (VR), and edge computing.

The synthesis aimed to present a balanced perspective on the current state of AI integration in PACS, highlighting both the benefits and the barriers to widespread adoption.

## 3 THE INTERSECTION OF AI AND PACS

### 3.1 Technological Foundations

Artificial intelligence (AI) is an interdisciplinary field described by John McCarthy as “*the science and engineering of making intelligent machines*” [26]. AI combines statistical algorithms and artificial neural networks (ANNs) to generate insights for decision-making and problem-solving in various contexts. ANNs are inspired by the biological neural networks of the human brain, allowing computer systems to “learn” complex relationships from data. Unlike traditional regression models, ANNs exhibit excellent fault tolerance, high processing speed, and the ability to model complex nonlinear relationships, making them well-suited for applications in medical imaging [27, 28].

### 3.2 Artificial Neural Networks (ANNs): Training and Behavior

The effectiveness of ANNs depends on a thorough training process, where the network's parameters are adjusted to optimize the weights of connections. This process, known as training, involves exposing the network to input data until it learns to respond correctly, reaching a state of convergence. ANNs consist of three primary layers: input, hidden, and output layers [28]. The perceptron, introduced by Frank Rosenblatt in 1958, is a basic single-layer model using supervised learning to classify input data into two categories [29]. However, it struggles with non-linear data, such as complex medical images.

The Backpropagation algorithm, a multilayer perceptron model, extends the single-layer perceptron by adding hidden layers, allowing it to handle more complex relationships. However, this model requires significant computational resources and depends heavily on the quality of the training data.

To handle more complex patterns, the backpropagation algorithm, a multilayer perceptron model, extends the single-layer network by adding hidden layers. This architecture allows it to capture intricate relationships but requires extensive computational resources and high-quality training data. Machine learning (ML), a subset of AI, leverages ANNs for predictive analytics and decision-making, typically categorized into three types: supervised, unsupervised, and reinforcement learning [30].

1. *Supervised Learning*: This method pairs input data with corresponding output labels. The network learns by adjusting weights based on the error between predicted and actual outcomes, making it highly effective for tasks such as image classification.
2. *Unsupervised Learning*: In this approach, the network groups input data based on inherent statistical properties without predefined output labels. It is useful for discovering hidden patterns in medical imaging data.
3. *Reinforcement Learning*: This technique involves a model learning through interactions with its environment, receiving feedback in the form of rewards or penalties. It is particularly beneficial for dynamic decision-making in medical imaging processes.

### 3.3 Deep Learning and Convolutional Neural Networks (CNNs)

Deep learning (DL), a subset of ML, focuses on learning complex data representations through multiple layers [31]. The most prominent deep learning model in medical imaging is the convolutional neural network (CNN), designed for processing image data. CNNs consist of three main layers: convolutional, pooling, and fully connected layers. The convolutional layer extracts features from the input image, the pooling layer reduces dimensionality, and the fully connected layer maps these features to classification outputs [32, 33].

CNNs have proven effective in detecting, segmenting, and classifying medical images, significantly improving diagnostic accuracy. For example, CNNs in MRI analysis have been used to detect brain lesions, outperforming traditional radiological methods in identifying early-stage tumors. However, deep learning models require extensive training on large, annotated datasets, which can be challenging to obtain in medical settings due to data privacy concerns [34, 35].

### 3.4 Integration of DICOM Standards in AI-Powered PACS

The DICOM (Digital Imaging and Communications in Medicine) standard, established in the 1980s, plays a crucial role in ensuring the interoperability of medical imaging systems. PACS, designed to manage and store medical images, relies heavily on DICOM standards for data generation, transmission, and display [1]. The integration of AI with PACS requires adherence to these standards, allowing for seamless communication between various imaging devices, servers, and diagnostic workstations.

The combined use of AI and DICOM standards has enabled the development of advanced PACS functionalities, such as automated tissue segmentation and multi-source data integration. These capabilities enhance diagnostic accuracy and streamline workflows, making PACS a vital component of modern radiology services [3].

### 3.5 Current State of AI-Powered PACS

AI-powered PACS have evolved beyond basic image storage solutions to become sophisticated diagnostic platforms capable of real-time image analysis and enhanced data management. The integration of AI models into PACS has enabled advanced functionalities such as automated lesion detection, multi-modal image fusion, and predictive analytics, significantly enhancing the diagnostic capabilities of medical imaging systems [6, 9].

Modern AI-enhanced PACS systems utilize deep learning algorithms for tasks including image reconstruction, segmentation, and classification. For example, convolutional neural networks (CNNs) are employed to process and analyze medical images, improving accuracy in detecting pathologies like brain tumors, pulmonary nodules, and liver lesions. The integration of deep learning techniques has accelerated image processing workflows, allowing for faster interpretation and reducing radiologist workload (Table **2**).

AI applications in medical imaging modalities such as MRI, CT, and ultrasound have shown significant advancements. In MRI, deep learning algorithms have been instrumental in noise reduction and motion artifact correction, which enhance image clarity and diagnostic precision [36]. Shimron and Perlman reviewed the latest advances in leveraging AI to improve MRI workflows [37]. Additionally, Yepes *et al*. [38] developed automatic methods to decrypt and uncompress MRI images at the voxel level to prevent disruptions in PACS operations. AI-enhanced CT image reconstruction can improve image resolution, providing finer anatomical details and enabling the use of lower radiation doses [39]. Moreover, AI-based automated tissue segmentation is becoming essential to the medical community, reducing the manual post-processing time of CT datasets, which is often time-consuming and subjective [40].

Similarly, AI techniques applied in CT imaging have optimized scan protocols, allowing for reduced radiation doses while maintaining high-resolution image quality. Ultrasound imaging has also benefited from AI integration, with automated tools for organ segmentation and real-time anomaly detection, facilitating faster clinical assessments [41].

AI also plays a key role in optimizing radiological workflows. Applications include motion artifact and noise reduction, automated radiation dose estimation, and generating MRI sequences based on findings. Additionally, AI contributes to workflow management by enhancing patient scheduling, prioritizing worklists, assisting with pre-dictation, extracting multi-source data, and supporting annotation-based feature extraction tasks and radiology reporting [42, 43].

AI-powered PACS systems have also improved workflow efficiency by automating routine tasks like image annotation, prioritization of critical cases, and structured reporting. These enhancements not only streamline radiological operations but also contribute to better clinical decision-making and patient management. The automation of report generation using natural language processing (NLP) tools in PACS has further reduced turnaround times, allowing radiologists to focus on complex cases.

Table **2** summarizes key advancements in AI-based integrated PACS, highlighting its role in enhancing diagnostic workflows across different medical specialties. These innovations illustrate the ongoing transformation of PACS from traditional image repositories to dynamic, AI-driven diagnostic tools that support precision medicine and personalized healthcare.

## 4 CHALLENGES AND BARRIERS TO AI-PACS INTEGRATION

Despite the promising potential of AI as a diagnostic tool, its adoption in PACS has been slow and limited. This is largely due to challenges associated with integrating AI into the existing radiology workflow and automating complex image management tasks. AI technologies in medical imaging still face significant limitations, including high false-positive rates and a limited capacity for complex reasoning.

Effective AI integration into PACS requires automation of key image management processes such as storage, retrieval, and distribution. AI systems must also support real-time image analysis, facilitating personalized treatment planning and enhancing diagnostic efficiency. Additionally, successful integration necessitates components for operationalization, performance monitoring, and continuous system improvement. AI tools must allow radiologists to evaluate the results, identify issues with image quality, and ensure reliability [44].

### 4.1 Data Privacy and Security

The storage server is a critical component of PACS, as server failures can severely disrupt radiology workflows and hinder automated image management. Ensuring the availability, integrity, and long-term storage of medical imaging data poses substantial challenges. Apart from managing large data volumes, it is crucial to safeguard the integrity and security of the information [45].

Data security in PACS systems is governed by the ISO/IEC 27000 standard, which defines the C-I-A triad: Confidentiality, Integrity, and Availability. Confidentiality ensures that only authorized personnel can access sensitive data. Integrity involves preserving the accuracy and completeness of data, often through the use of verification codes that detect any alterations. Availability ensures that authorized users have continuous access to the data, supported by backup systems and redundancy measures [46].

Adhering to the C-I-A triad is essential for PACS systems, which should also implement data redundancy via backup databases or secondary storage servers to prevent data loss. Regenerating lost data can be time-consuming, especially when manual migration of archived images is required [47, 48]. Table **3** outlines recommended measures for enhancing PACS information security, crucial for mitigating insider threats and ensuring data protection during disaster events [49].

### 4.2 Regulatory and Compliance Issues

Regulatory and legal challenges in the context of PACS have not been thoroughly addressed, despite their importance. It is vital to consider the protection and security of sensitive patient data, as well as the legal admissibility, validity, and reliability of digital images. Key issues include data retention periods, system upgrades, and the impact of image compression. For instance, lossless compression preserves image quality, whereas lossy compression may result in degradation. Until regulatory standards are fully established, the DICOM committee and the ECRI organization recommend using lossless images to maintain diagnostic accuracy [50].

PACS is classified as a Class 2 medical device, with specific regulatory requirements varying by country. In the United States, for example, PACS components may require FDA 510(k) premarket notification [51]. Certain elements of PACS may be subject to additional regulations depending on their functions, while others may fall under less stringent controls.

Part 15 of the DICOM standard (PS 3.15) outlines security profiles for communication and digital signatures, addressing licensing, staff accreditation, and technical responsibilities. HIPAA regulations further ensure the integrity, authenticity, and confidentiality of data, especially when image retrieval extends beyond a protected local area network (LAN). Personalized digital signatures are recommended to secure data transmission in these instances.

To comply with legal and ethical requirements, PACS systems and their integrated AI tools must adhere to established standards like DICOM and HIPAA, as well as broader information technology guidelines. Organizations must implement controls based on their specific policies and regulatory frameworks [52].

### 4.3 Interoperability and Standardization

Accessing and sharing digital health data is challenging due to heterogeneous data formats, the sensitivity of the information, and the need for strict privacy and security measures. Interoperability in medical imaging refers to the secure and timely integration of health information systems, which is essential for complete and accurate clinical data interpretation. A lack of interoperability can result in fragmented data, leading to suboptimal clinical outcomes and increased costs.

Achieving interoperability is crucial for the integration of technologies from different PACS vendors, especially when handling diverse data structures and various media formats, including static and dynamic images, 2D and 3D images, as well as DICOM and non-DICOM data. Security and privacy are fundamental considerations, and integrating AI tools with PACS to enhance efficiency must address these concerns, such as by automating study or series selection [53].

Standards play a key role in promoting interoperability. PACS systems should support industry standards like DICOM and HL7 to enable the seamless exchange of information with other healthcare systems, including HIS and RIS, as well as with medical imaging devices. The evolution of the HL7 framework into the Fast Healthcare Interoperability Resources (FHIR) standard has improved the flexibility of healthcare data exchange. FHIR combines the best features of previous HL7 standards, providing a unified specification while leveraging modern web technologies for rapid deployment and ease of adoption [54].

## 5 INFRASTRUCTURE AND WORKFORCE READINESS FOR AI-PACS INTEGRATION

The integration of AI into PACS offers numerous advantages, including:

1. Enhanced capability to process large datasets.
2. Functioning as an effective second reader, reducing diagnostic errors.
3. Lower risk of missing relevant findings.
4. Shorter diagnostic times.
5. Automation of image management tasks.

Manufacturers have begun embedding intelligent tools within PACS to boost diagnostic accuracy and streamline radiological workflows. These enhancements include features like voice dictation, automated report generation, and the use of prior reports to prepopulate templates, aiding in the assessment of disease severity. AI integration also supports a unified worklist that automates the assignment of exams based on body regions, templates, protocols, and communication of results, all conforming to international standards.

### 5.1 Infrastructure Readiness

To meet the demands of modern radiology, AI tools have been integrated into both hardware and software infrastructures. This includes advanced workload management solutions, robust visualization software, cloud computing resources, and AI applications for improved workflow management. Future developments may incorporate virtual and augmented reality to provide immersive visualization of medical images.

With increasing interoperability and interconnected health data systems, advancements in machine learning algorithms and big data analytics continue to enhance medical imaging processes. The widespread adoption of cloud connectivity has improved patient care by streamlining data processing and enhancing the efficiency of medical imaging workflows.

Software solutions like intelligent worklists have been developed to optimize productivity, alleviate “list anxiety,” and accelerate patient care. These tools utilize real-time data to identify potential bottlenecks and suggest corrective actions, helping healthcare staff minimize delays and reduce hospital stays. Machine learning applications across various modalities have also facilitated the integration of heterogeneous data sources, including medical images, lab results, and electronic health records.

Hospitals and medical research centers must ensure they have the appropriate IT infrastructure to support these advancements. For example, edge computing sites offer local data processing capabilities, reducing latency and supporting enhanced workflow management in healthcare settings.

### 5.2 Workforce Adaptation and Training

The integration of AI into diagnostic imaging is transforming the field by automating tasks and assisting in anomaly detection. Radiologists must be adequately trained to use these tools effectively and understand their implications for medical practice and radiology education.

Radiology departments should leverage AI as an educational tool for trainees while also recognizing its limitations. This balanced approach will help prepare the next generation of radiologists to integrate AI into their daily workflow and make informed clinical decisions.

Active collaboration between radiologists and AI developers is crucial throughout the AI lifecycle-from design and development to deployment. This partnership ensures that AI tools are designed to meet clinical needs and minimize potential risks in the decision-making process.

To support this transition, the Radiological Society of North America (RSNA) offers an Imaging AI Certificate Program [54]. This program provides radiologists with essential knowledge to address the challenges posed by AI in medical imaging, including model development, fairness assessment across diverse populations, and navigating the complexities of AI processes like data input, pre-processing, feature extraction, and classification.

## 6 CLINICAL APPLICATIONS AND CASE STUDIES OF AI IN PACS

The integration of artificial intelligence (AI) with Picture Archiving and Communication Systems (PACS) has shown promising results across various clinical domains. Multiple studies have demonstrated AI's ability to enhance diagnostic accuracy, streamline workflows, and support personalized medicine by leveraging predictive analytics and integrating with electronic health records (EHRs). Reviews by Khalifa and Najjar emphasize AI’s transformative role in radiology, particularly in image segmentation, computer-aided diagnosis, and workflow optimization [55, 56].

The following case studies highlight the significant benefits and advancements achieved through successful AI-PACS integration in clinical practice.

### 6.1 Successful Integrations of AI with PACS

#### 6.1.1 Pneumonology

AI has proven particularly effective in pneumology, aiding in the interpretation of chest radiographs and prioritizing diagnostic workflows. AI-driven diagnostic reports help referring physicians make timely decisions, while automated worklist prioritization improves radiologists' efficiency [57]. A streamlined reporting workflow integrating AI results into structured radiology reports has become essential for enhancing diagnostic accuracy and speed [58]. Fig. (**3**) illustrates the complete AI-to-SR pipeline workflow.

In one notable study, Aidoc's AI software was evaluated for prioritizing acute intracranial hemorrhage (ICH) cases in a dataset of 8,723 non-contrast head CT scans. The AI flagged 1,829 scans as positive, reducing review delays by 90% for outpatients (604 minutes) and 10% for inpatients (38 minutes). This substantial decrease in review time significantly enhanced diagnostic efficiency at an academic medical center [59].

A retrospective analysis assessed an AI algorithm’s performance in detecting pulmonary embolism (PE) on 1,504 contrast-enhanced CT scans from COVID-19 patients. The AI demonstrated a sensitivity of 93.2% and specificity of 99.6%, maintaining high accuracy across all severity levels. The algorithm was more effective on CT pulmonary angiography than standard contrast-enhanced CT scans, showcasing its ability to enhance diagnostic precision regardless of disease severity [60].

Another case study evaluated Aidoc's FDA-approved convolutional neural network (CNN), C-spine, for detecting cervical spine fractures on 665 CT examinations. The CNN achieved an accuracy of 92%, with 76% sensitivity and 97% specificity, compared to radiologists who achieved 95% accuracy, 93% sensitivity, and 96% specificity. The CNN’s ability to prioritize worklists and assist in detecting cervical spine fractures highlights its potential as a supportive tool for radiologists [61].

#### 6.1.2 Neurology

AI integration has shown remarkable promise in neurology, particularly in detecting intracranial hemorrhages (ICH) and primary brain tumors. A multi-center cohort study assessed the effectiveness of AI in identifying ICH on 4,946 non-contrast head CT scans at level I trauma centers. Using Aidoc's software, a neuroradiologist reviewed discrepancies between AI predictions and radiology reports. The AI detected 29 additional ICH cases that human radiologists missed, improving detection rates by 12.2% and reducing the incidence of missed ICHs from 10.9% to 1.9%. This study underscores the potential of AI to significantly enhance diagnostic accuracy in emergency settings [62].

A retrospective analytical study investigated AI’s role in detecting primary brain tumors in pediatric patients using MRI images. Machine learning algorithms applied through artificial neural networks achieved high accuracy in identifying tumors in this population, demonstrating the value of AI in pediatric neuroradiology [63].

Further research has explored the use of AI integrated with PACS for detecting neurodegenerative conditions. A notable study from the Stevens Institute of Technology developed an AI system utilizing convolutional neural networks, achieving a 96% success rate in identifying early stages of Alzheimer’s disease. This application of AI in neuroimaging represents a significant advancement in the early diagnosis and management of neurodegenerative diseases [64].

### 6.2 Innovative AI Applications in Medical Imaging

The integration of advanced artificial intelligence (AI) applications in medical imaging has the potential to revolutionize the field, enhancing the accuracy, efficiency, and reliability of diagnostic analyses. Recent reviews highlight significant advancements in AI technologies, particularly in natural language processing (NLP) and image analysis. For instance, Bidirectional Encoder Representations from Transformers (BERT), an NLP model, has transformed radiology by effectively classifying and extracting complex information from radiology reports. BERT’s ability to analyze sentence context by considering both preceding and following words has improved protocol assignment and the interpretation of imaging studies [65].

NLP innovations have facilitated the automated understanding of clinical narratives within radiology reports, enabling better protocol assignment and more accurate image interpretation. These advancements streamline clinical workflows, enhance patient care, and address challenges such as reproducibility and explainability, fostering greater collaboration in radiology practice [66].

The following examples illustrate key innovative AI applications in medical imaging:

#### 6.2.1 Liver and Lesion Segmentation

Skwirczyński *et al*. utilized the nnU-Net framework for segmenting liver and lesions in MR images, achieving a Dice coefficient of approximately 0.98 for liver segmentation and an AUC ROC of 0.925 for lesion classification. These results demonstrate nnU-Net’s potential to assist in clinical diagnosis by providing reliable automated analysis [67].

#### 6.2.2 Enhanced Liver Diffusion-Weighted Imaging (DWI)

An AI algorithm was developed to mitigate motion-induced signal loss in liver DWI caused by cardiac and respiratory movements. Deep learning techniques guided post-processing, improving liver homogeneity and lesion detectability, thus offering significant clinical benefits [68].

#### 6.2.3 Early Breast Cancer Detection

AI networks analyzing MRI scans of BRCA mutation carriers significantly improved early detection of breast cancer, correctly identifying 65% of tumors initially missed by radiologists. This approach holds promise for earlier diagnosis, potentially improving survival rates and patient quality of life [69].

#### 6.2.4 Hybrid Diagnostic Approach for Breast Cancer

A study combining automated feature extraction with domain expertise achieved up to 98% accuracy in distinguishing benign from malignant cases using neural network classifiers. This hybrid method enhanced diagnostic performance using histopathology images [70].

#### 6.2.5 Lung Cancer Detection from Histopathological Images

AI applications improved the early detection and classification of lung cancer, achieving 91.57% accuracy with a Decision Tree classifier and Grey Wolf Optimization. This approach enhanced segmentation, feature extraction, and classification capabilities [71].

#### 6.2.6 Improved Lung Field Segmentation

A method combining superpixel resizing with encoder-decoder networks significantly enhanced lung field segmentation accuracy in chest X-rays, aiding in the diagnosis of pulmonary diseases such as tuberculosis, pneumonia, and lung cancer [72].

#### 6.2.7 Prostate MRI Segmentation

The nnU-Net model demonstrated superior performance in automating prostate MRI segmentation, achieving high accuracy and lower error rates. This is crucial for accurate diagnosis and effective treatment planning [73].

#### 6.2.8 Tumor Volume Measurement in Pediatric Oncology

The nnU-Net framework was also used to automate tumor volume measurements in pediatric oncology, achieving a median Dice score of 0.90. This score closely matched manual segmentations, indicating high accuracy and reliability [74].

#### 6.2.9 Synthetic MRI Image Generation

An AI-driven open-source tool was developed to generate synthetic MRI images, enhancing diagnostic imaging efficiency and providing educational opportunities with a user-friendly, customizable platform [75].

#### 6.2.10 Denoising Micro CT Images

The UnetU model, based on the U-net CNN architecture, significantly improved the quality and speed of denoising Micro CT images, achieving a 15-fold faster performance compared to traditional methods [76].

#### 6.2.11 Generative Adversarial Networks (GANs) in Tomographic Imaging

A study utilizing GANs to infer missing measurements in tomographic imaging showed improved image quality, reducing artifacts by up to 7 dB in Peak Signal-to-Noise Ratio. GANs, consisting of a generator and a discriminator, collaborate to enhance image quality [77].

### 6.3 AI Applications Using MRI Medical Imaging

#### 6.3.1 Recent Advances in AI Applications for Pelvic MRI

Artificial intelligence (AI) has significantly advanced the field of pelvic magnetic resonance imaging (MRI), especially in diagnosing conditions affecting the prostate, bladder, uterus, ovaries, and rectum. AI techniques, including machine learning (ML) and deep learning (DL), have enhanced various stages of the pelvic MRI diagnostic process, such as image acquisition, reconstruction, lesion detection, and risk assessment [78].

One notable improvement is the automation of field-of-view (FOV) settings based on anatomical structures, leading to better image angulation and enhanced scan quality [79, 80]. Deep learning reconstruction (DLR) has reduced scan times by up to 70% while maintaining or even enhancing the quality of T2-weighted imaging (T2WI) [81-84]. The reduction of noise and artifacts in these scans has greatly improved diagnostic accuracy.

AI has also made significant strides in lesion detection and segmentation. Advanced models now generate heat maps to pinpoint potential lesion sites, while automated segmentation tools facilitate quantitative evaluations of tumor volume and parameters like the apparent diffusion coefficient (ADC) [85-87]. In prostate MRI, AI applications have shown notable improvements in detecting and classifying clinically significant cancers, aiding in more precise diagnosis and treatment planning [88-90].

Despite these advances, challenges persist. A key issue is the generalizability of AI models, which are often trained on limited datasets, making them vulnerable to overfitting. Researchers suggest that using large, multicenter datasets could enhance model robustness [91]. Additionally, increasing the interpretability of AI algorithms remains essential for gaining clinical acceptance and validation [92-94].

#### 6.3.2 Diagnostic Performance of AI-based Algorithms in Discriminating Multiple Sclerosis Using MRI Features

AI algorithms have shown promise in differentiating Neuromyelitis Optica Spectrum Disorder (NMOSD) from Multiple Sclerosis (MS) using MRI features. Accurate discrimination between these conditions is crucial due to their overlapping clinical and radiological characteristics, which can lead to diagnostic errors and inappropriate treatment [95, 96]. Both ML and DL models have been employed to enhance diagnostic accuracy, particularly in analyzing brain and spinal MRI scans [97, 98].

A meta-analysis of 15 studies revealed that AI-based algorithms demonstrated strong diagnostic performance, with pooled accuracy, sensitivity, and specificity of 82%, 83%, and 80%, respectively [99]. Notably, MRI-based algorithms alone achieved similar diagnostic metrics, with an accuracy of 83%, sensitivity of 81%, and specificity of 84%, underscoring AI’s potential in improving diagnostic precision [100]. Distinctive MRI features, such as Dawson’s finger-type lesions for MS and linear ependymal lesions for NMOSD, are commonly utilized by AI models to enhance diagnostic accuracy [101].

However, variations in MRI imaging protocols and inconsistencies in AI model performance across studies present challenges. Future research should prioritize the use of multicenter datasets and standardized imaging protocols to enhance the generalizability and clinical reliability of AI algorithms [95]. Additionally, integrating multimodal data, such as combining clinical information with imaging features, may further improve the diagnostic capabilities of AI systems [102].

#### 6.3.3 AI Applications in Breast Imaging

AI has made a significant impact on breast imaging, especially in screening mammography for breast cancer detection. Early computer-aided detection (CAD) systems faced limitations, but recent advancements in ML and DL have demonstrated improved diagnostic performance [103]. Currently, there are over 20 FDA-approved AI applications for breast imaging, focusing on tasks such as cancer detection, decision support, breast density quantification, and workflow optimization [103].

AI has shown notable value in breast cancer detection during screening mammography. AI algorithms trained on extensive datasets have achieved sensitivity and specificity levels comparable to those of radiologists, with the potential to reduce both false positives and false negatives [104]. These enhancements allow AI models to be seamlessly integrated into clinical workflows, either as a second reader or as a triage tool, improving diagnostic efficiency. Additionally, AI has proven beneficial in reducing the time radiologists spend interpreting digital breast tomosynthesis (DBT) images, thereby enhancing overall productivity [105, 106].

Beyond cancer detection, AI applications are expanding to other areas of breast imaging, such as risk assessment and evaluating treatment responses. AI-based tools for breast density quantification have shown high accuracy and are now routinely used in clinical practice to aid in risk stratification [107]. Furthermore, AI is being explored for assessing responses to neoadjuvant chemotherapy, with promising results in predicting treatment outcomes based on MRI features [108].

Despite these advances, challenges persist in generalizing AI models across diverse populations and imaging systems. Ensuring the robustness of AI applications in various clinical settings is a priority for future research to maintain consistent performance and reliability [109].

#### 6.3.4 Ethical Challenges of Artificial Intelligence in Neuroradiology

The integration of AI into neuroradiology has the potential to enhance diagnostic accuracy, support clinical decision-making, and enable personalized treatment plans [110]. However, these advancements also bring forth significant ethical challenges, particularly related to data privacy, informed consent, and liability [111]. AI models require large datasets, often derived from anonymized patient records, which raises concerns about data ownership and control. While some argue that anonymized data should be freely accessible for research, others emphasize the need for securing patient consent and trust in data-sharing practices [112-114].

Data privacy is a critical concern, especially in neuroradiology, where AI models could inadvertently expose personal identifiers. Techniques like defacing and skull-stripping are employed to protect patient identities, but advanced imaging methods may still enable re-identification based on surface anatomy features [115, 116]. This highlights the need for stringent data governance protocols that balance ethical considerations with the necessity of using medical data for AI research. Privacy regulations and concerns also differ across jurisdictions, complicating the establishment of universal guidelines [112].

Bias in AI algorithms is another significant issue. When AI systems are trained on datasets that lack diversity, they may yield biased outcomes that disproportionately affect underrepresented groups. This has been observed in medical imaging, where algorithms trained on imbalanced datasets have demonstrated reduced diagnostic accuracy for certain demographic populations [117-119]. Addressing this problem requires prioritizing dataset diversity during model development to prevent exacerbating health disparities [120, 121].

Liability is a complex challenge in the context of AI-supported clinical decision-making. Determining responsibility for errors made by AI systems can be contentious-should liability rest with the healthcare providers who use the AI tools or with the developers who create them? Some studies suggest shared responsibility between clinicians and developers, but this remains a debated issue that requires further legal clarification [122-124].

While AI offers significant potential benefits for neuroradiology, addressing the ethical issues of data privacy, bias, and liability is essential for responsible and equitable deployment of these technologies in clinical practice [125-127].

#### 6.3.5 AI-Enhanced Detection of Clinically Relevant Anomalies in MRI

AI has transformed the detection of clinically relevant anomalies in magnetic resonance imaging (MRI), significantly increasing both diagnostic speed and precision. The integration of advanced ML and DL techniques has facilitated the identification of structural and functional abnormalities that were previously difficult to detect using traditional methods [20].

Anomalies in MRI, such as tumors, infarcts, and degenerative changes, represent deviations from typical anatomical patterns. AI systems excel in processing large datasets and extracting intricate features, often surpassing the capabilities of manual detection. The shift towards explainable AI (XAI) models has further enhanced clinical transparency, enabling clinicians to understand the rationale behind AI-generated diagnoses, which is critical for building clinical trust and promoting widespread adoption [128].

AI-based anomaly detection in MRI typically involves preprocessing steps like image normalization and denoising to enhance diagnostic clarity [100]. Convolutional neural networks (CNNs), a type of DL model, have been particularly effective in segmenting and classifying abnormalities, including tumors and vascular anomalies [98]. CNNs have shown high sensitivity and specificity in identifying brain lesions, outperforming conventional radiological approaches [97].

However, challenges remain, particularly in ensuring the generalizability of AI models. Many models are trained on limited or homogeneous datasets, which can compromise their performance in diverse clinical environments. To address this, robust model development requires multicenter datasets and continuous learning to enhance diagnostic reliability across various settings [20].

The field of AI-enhanced anomaly detection in MRI is evolving rapidly, with the potential to greatly improve diagnostic accuracy and efficiency. Nonetheless, ongoing research and development are necessary to overcome issues related to data diversity and model interpretability [20].

#### 6.3.6 AI-Enabled Prospects in MRI-Guided Radiotherapy

MRI-guided radiotherapy (MRIgRT) marks a significant advancement in radiation oncology by providing superior anatomical detail through continuous imaging during treatment [129]. This approach allows precise targeting of tumors while minimizing damage to surrounding healthy tissues [130]. One of the most promising applications of AI in MRIgRT is the enhancement of real-time motion management. AI algorithms, especially deep learning models, are being developed to predict and compensate for intra-fractional tumor motion, which is crucial for achieving optimal treatment outcomes [131, 132].

Current AI applications in MRIgRT include motion tracking, estimation, and prediction. Using cine MRI sequences, AI can localize treatment targets in real-time and anticipate movements caused by respiration or other physiological factors [51]. This predictive capability is vital for reducing system latency, significantly influencing the accuracy of beam delivery during radiotherapy [133, 134]. Deep learning models, such as convolutional neural networks (CNNs) and long short-term memory (LSTM) networks, have shown strong potential in motion prediction, consistently outperforming traditional methods [135, 136]. These AI-driven models enhance the real-time adaptation of radiotherapy beams, ensuring accurate dose delivery even during complex tumor movements [137, 138].

Despite these advancements, integrating AI into clinical practice remains challenging, particularly due to the need for larger, more diverse datasets to improve the generalizability of AI models [50]. Continued development of tailored AI solutions for MRIgRT is expected to enhance the precision of radiation therapy, reduce treatment durations, and ultimately improve patient outcomes [139].

#### 6.3.7 The Role of AI in Prostate MRI Quality and Interpretation

AI has made substantial contributions to improving the quality and interpretation of prostate MRI, particularly in multiparametric MRI (mpMRI), which includes T2-weighted imaging (T2WI), diffusion-weighted imaging (DWI), and dynamic contrast-enhanced MRI (DCE-MRI). These imaging modalities are essential for detecting and managing prostate cancer (PCa), but variability in acquisition and interpretation remains a challenge due to differences in scanner technologies, imaging parameters, and observer variability [140, 141]. Although efforts to standardize prostate MRI using systems like PI-RADS and PI-QUAL have improved consistency, human error persists [142].

AI integration into prostate MRI aims to automate processes and reduce errors, particularly in tasks like image quality assessment, registration, segmentation, and feature extraction. AI-based tools have shown improvements in diagnostic performance by enhancing image consistency across various clinical settings [143, 144]. The ability of AI algorithms to process large datasets and reduce variability between institutions highlights their potential for standardizing image quality and interpretation [145].

However, the clinical implementation of AI in prostate MRI faces challenges, including the need for rigorous validation and external testing. Variability in MRI quality across centers underscores the necessity for AI systems capable of adapting to different imaging protocols and scanner types [146]. Additionally, reliance on annotated datasets introduces subjectivity, which must be mitigated through robust reference standards [147]. While AI shows promise for enhancing prostate MRI quality and interpretation, further research is required to ensure its effectiveness in diverse clinical environments [148].

#### 6.3.8 AI in Predicting Survival for Brain Tumor Patients

Artificial intelligence (AI) has become a valuable tool for predicting survival outcomes in brain tumor patients using MRI data. Gliomas, particularly glioblastomas (GBM), pose significant prognostic challenges due to their aggressive nature and heterogeneity [21]. GBM, the most common and lethal form of glioma, has a poor prognosis, with only 5-7% of patients surviving beyond five years despite intensive treatment [149, 150].

AI-based techniques, including ML and DL algorithms, have demonstrated success in predicting overall survival (OS) by extracting quantitative data from MRI images through radiomics. Radiomic analysis enables the classification of patients into survival groups (short-, mid-, and long-term), with ML models like support vector machines (SVMs) and random forests (RFs) achieving accuracy rates up to 98% [151, 152]. DL models, which learn automatically from large datasets without hand-crafted features, are emerging as superior tools for survival prediction [153].

Recent AI models have focused on leveraging multimodal MRI data, including perfusion-weighted imaging (PWI) and diffusion-weighted imaging (DWI), to enhance the accuracy of survival predictions. These techniques provide comprehensive information about tumor physiology, contributing to more robust predictive models. Nonetheless, the generalizability of these AI models remains limited by the small and non-diverse datasets used for training [154, 155]. While AI shows potential in predicting survival for brain tumor patients, broader validation on diverse datasets is necessary for clinical applicability [21].

#### 6.3.9 Innovations in AI for Cardiac MRI: Current and Future Prospects

AI is revolutionizing cardiac MRI, enhancing every stage of the imaging process, from acquisition to analysis. Cardiac MRI is a vital tool for diagnosing and managing cardiovascular diseases, but its complexity and lengthy acquisition protocols have historically limited its widespread clinical adoption [156]. Recent advances in AI, particularly in DL and ML, have streamlined image acquisition, reconstruction, and post-processing, significantly reducing scan times and improving diagnostic precision [157].

CNNs are frequently employed in cardiac MRI for tasks such as image segmentation and motion tracking. AI also facilitates automated prescription and parameter optimization, effectively transforming MRI scanners into “self-driving” systems that require minimal manual input [158, 159]. This shift results in faster scans and reduces patient discomfort by minimizing breath-hold requirements. DL-based techniques are also enhancing image reconstruction, removing artifacts from undersampled data and yielding clearer, higher-quality images [160, 161].

AI extends beyond efficiency gains to provide advanced diagnostic insights through methods like radiomics and DL feature extraction. These approaches allow AI models to detect novel imaging biomarkers that may be imperceptible to human readers, improving prognosis and supporting personalized treatment strategies [162]. The integration of imaging data with clinical, genetic, and wearable device information has the potential to create comprehensive diagnostic tools for precision cardiovascular care [163].

However, challenges remain, including the need for extensive and diverse datasets to develop robust AI models that can generalize across different patient populations and imaging systems [164]. Additionally, concerns about algorithmic bias and the interpretability of AI models must be addressed before AI can be fully integrated into routine clinical practice. As AI continues to evolve, future innovations will likely focus on enhancing model robustness and expanding the role of AI in personalized medicine [165]. Table **4** provides a summary of the latest AI innovations using MRI across various medical specialties.

#### 6.3.10 Clinical Impact of CNNs and NLP in PACS Integration

##### 6.3.10.1 Convolutional Neural Networks (CNNs) in Medical Imaging

**Enhanced Diagnostic Accuracy**: CNNs have demonstrated significant improvements in diagnostic accuracy for various medical imaging tasks. For instance, they have been effectively applied in the detection and classification of lung nodules from chest X-ray images, aiding in early diagnosis and treatment planning [166].

**Workflow Optimization**: The application of CNNs in medical image analysis has streamlined diagnostic processes, reducing the time required for image interpretation and thereby improving clinical workflow efficiency [167].

##### 6.3.10.2 Natural Language Processing (NLP) in Radiology Reporting

**Structured Information Extraction**: NLP techniques have been pivotal in extracting structured information from unstructured radiology reports, facilitating better data management and retrieval. This advancement supports clinical decision-making and enhances patient care [66].

**Automated Report Generation**: NLP has enabled the development of systems that can automatically generate structured radiology reports from free-text inputs, improving report consistency and reducing the potential for errors [168].

## 7 ETHICAL IMPLICATIONS AND PATIENT-CENTERED INSIGHTS ON AI-PACS

### 7.1 Ethical Implications

As of late 2023, the U.S. Food and Drug Administration (FDA) had approved over 340 artificial intelligence medical devices (AIMDs) in medical imaging [169]. While the advantages of AI integration in healthcare have been widely discussed, there are critical ethical risks that need consideration. These risks include issues of confidentiality, algorithmic opacity, clinician deskilling, and fairness [170]. Mike Stephen *et al*. analyzed the ethical challenges of AI in healthcare and proposed frameworks for responsible AI implementation [171]. This section explores the ethical implications of integrating AI with PACS, using the four principles of medical ethics: nonmaleficence, beneficence, autonomy, and justice.

### 7.2 Ethical Implications

#### 7.2.1 Nonmaleficence and Beneficence

The ethical principles of nonmaleficence (avoiding harm) and beneficence (promoting well-being) require that AI medical devices (AIMDs) provide clear benefits without causing harm. Developers and regulators must ensure that claims about AIMD performance are well-founded, and clinicians must prioritize patient safety when using these tools [172].

Three main considerations have been identified for clinician responsibility. First, AIMD systems should only be used for their intended purposes, especially in disease detection and diagnosis. Improper use could obscure critical features in medical images or provide overly simplistic binary outputs, potentially misleading clinicians. Second, clinicians need to understand the characteristics of the populations on which AIMD systems were validated, as accuracy may vary if patient demographics differ significantly from the training data. Finally, clinicians must be cautious of automation bias-uncritical acceptance of AI-generated outputs-which can lead to errors in diagnosis and treatment [169].

Maximizing patient benefits while minimizing harm requires careful consideration of the trade-offs between false positives and false negatives. Clinicians and patients must be aware of potential errors to make informed decisions, balancing longevity with quality of life. AIMDs should not make rigid decisions on these trade-offs but instead allow flexibility based on individual patient preferences [173, 174]. Additionally, regulatory processes must account for variations in clinical settings. AIMDs developed in high-income environments may underperform in low-resource settings, affecting diagnostic accuracy. Continuous monitoring of AIMD performance after software updates or changes in patient populations is essential to maintain reliability [175].

#### 7.2.2 Patient Autonomy

Respecting patient autonomy involves ensuring that patients are fully informed about the risks, limitations, and benefits of AIMD interventions. Clinicians must communicate key parameters, such as diagnostic accuracy (*e.g.*, false positive and negative rates), to help patients understand the balance of risks and benefits [176].

A significant challenge with AIMDs is their lack of explainability [177]. Many clinicians may find it difficult to fully understand the mechanisms of complex AI systems, making it challenging to convey this information to patients. Additionally, to prevent proprietary data issues, certain decision-making patterns within AIMDs may lack transparency, further complicating patient education [178].

#### 7.2.3 Justice

There are growing concerns about biases in machine learning algorithms related to race, sex, and socioeconomic status [175]. It is crucial that clinicians and administrators ensure procedural fairness, treating patients equally regardless of characteristics like race, age, gender, or religion. In some cases, protected characteristics (*e.g.*, race or gender) may influence medical decisions, but their inclusion must be clearly justified to avoid perpetuating biases [169].

To address distributive fairness, developers have implemented strategies during data collection and model training to minimize bias. However, these efforts alone may not eliminate disparities in access to AI technologies or the risk of exacerbating structural inequalities within the healthcare system [169].

### 7.3 Patient-Centered Perspectives

Patient-centered care (PCC) emphasizes addressing individual healthcare needs and empowering patients to actively participate in their care [179]. Effective PCC involves respecting patient preferences, values, and needs and fostering shared decision-making between patients and healthcare providers [180]. Clear communication is crucial for enabling patients and their families to make informed decisions about their treatment options [181].

In the context of AI integration with PACS, the first step in ensuring PCC is to address the issue of explainability. Beyond technical details, explainability helps resolve potential discrepancies between AI outputs and clinical judgments, which may arise due to biases or errors in the AI system. However, the complexity of AI models often makes it challenging for clinicians to fully grasp and explain their decision-making processes to patients. Multidisciplinary collaboration involving data scientists and clinical experts can help bridge this gap and enhance understanding [182].

The next step is aligning AI-integrated PACS decisions with patient and family preferences, a key component of shared decision-making. Evidence-based tools have been developed to facilitate shared decision-making by presenting key facts about the patient’s condition, risks, and potential outcomes. These tools enable patients and clinicians to collaboratively choose the option that best aligns with the patient’s goals and expectations [183, 184].

Explainable AI systems can support PCC by making patients feel more informed and engaged in their care. By improving patients’ understanding of risks, these systems encourage active participation in shared decision-making, ultimately enhancing patient satisfaction and outcomes [182].

Addressing the ethical challenges of AI integration in PACS requires careful planning, transparency, and a commitment to patient-centered benefits. By establishing strong regulatory frameworks and prioritizing patient autonomy, justice, and beneficence, healthcare providers can harness the potential of AI to enhance clinical practice and improve patient outcomes.

## 8 FUTURE PERSPECTIVES: EMERGING TRENDS IN AI AND PACS

The future of PACS is set to incorporate more advanced AI integrations, significantly enhancing capabilities in predictive analytics and personalized medicine. As AI technology continues to evolve, PACS is expected to become increasingly intelligent, offering greater support for clinical decision-making and improving patient outcomes. The ongoing development of international standards, such as DICOM and Fast Healthcare Interoperability Resources (FHIR), will further enhance the interoperability of PACS with other healthcare systems globally, facilitating seamless data sharing and integration [6].

The integration of AI in medical imaging represents a transformative shift in diagnostic processes, providing substantial benefits in accuracy, efficiency, and patient care. The future of AI in medical imaging is poised for continued growth and innovation, with AI-driven tools becoming more sophisticated. These advancements are expected to enhance diagnostic support, reduce radiologists' workloads, and contribute to the advancement of personalized medicine, ultimately improving patient outcomes [18].

### 8.1 Emerging Technologies

Several emerging technologies are anticipated to shape the future landscape of PACS and AI integration. Notable advancements in deep learning algorithms are expected to enhance the precision of image analysis, while natural language processing (NLP) will play a key role in extracting clinical insights from radiology reports. NLP models, such as Bidirectional Encoder Representations from Transformers (BERT), have already demonstrated success in interpreting complex medical language, paving the way for more effective decision-support tools [57].

Augmented reality (AR) and virtual reality (VR) are poised to become valuable tools for radiologists, offering immersive visualization experiences for complex medical images. These technologies could enhance surgical planning and provide better anatomical understanding, particularly in training and education scenarios [11]. Edge computing is also gaining traction in healthcare, allowing for faster data processing at the site of data generation, such as within a PACS. This reduces latency and enables real-time AI-driven analysis. When combined with cloud computing, edge computing will enhance data accessibility, storage, and sharing, ultimately improving the efficiency and scalability of PACS systems [11].

### 8.2 Predictions and Expectations for PACS and AI

Looking ahead, AI-powered PACS are expected to incorporate more advanced predictive analytics tools capable of anticipating disease progression and assisting clinicians in making informed treatment decisions. These systems will likely support integrated multi-modal imaging, allowing simultaneous analysis of different imaging modalities-such as MRI, CT, and ultrasound-providing a more comprehensive diagnostic perspective [5].

AI will also play a critical role in workflow optimization by automating routine tasks, including image annotation, triage, and report generation. This automation will help reduce radiologists' workload, expedite the diagnostic process, and enhance overall healthcare efficiency, leading to better patient outcomes [5].

Future AI systems are expected to become more transparent, featuring explainable AI models that provide clinicians with insights into the reasoning behind AI-generated diagnoses. This transparency is vital for building trust in AI tools and ensuring their widespread acceptance in clinical practice. Explainable AI will also help address concerns related to algorithmic bias and improve the interpretability of AI-driven decisions [166].

The future of PACS and AI integration is marked by increasing sophistication and innovation. As AI technology advances, it will further enhance PACS capabilities, driving improvements in diagnostic accuracy, clinical decision-making, and patient care. By leveraging emerging technologies and aligning with evolving international standards, healthcare providers will be better equipped to meet the growing demands of modern medical practice [10]. Table **4** summarizes the recent AI innovations using MRI across various medical specialties, highlighting the key areas of impact and future potential.

#### 8.2.1 Broader Implications of AI Integration

The integration of artificial intelligence (AI) in PACS demonstrates not only advancements in medical imaging but also significant implications for the broader healthcare ecosystem. These findings emphasize the versatility and scalability of AI technologies in the following areas:

##### 8.2.1.1 Personalized Medicine

The ability of AI to analyze and integrate large-scale imaging data with electronic health records (EHRs) sets the stage for precision diagnostics and treatment planning. For instance, predictive analytics derived from AI-enhanced PACS could inform individualized treatment regimens by correlating imaging biomarkers with patient-specific clinical data.

##### 8.2.1.2 Healthcare Interoperability

By addressing challenges in data standardization and system integration within PACS, AI advancements contribute to broader interoperability efforts in healthcare. Seamless data exchange across diverse healthcare platforms could facilitate multidisciplinary collaboration, enhancing overall care coordination.

##### 8.2.1.3 Medical Education and Training

The automation of image interpretation and analysis creates opportunities for AI-powered learning platforms. These tools could provide medical trainees with real-time feedback, simulated case scenarios, and curated learning materials, accelerating the development of diagnostic expertise.

##### 8.2.1.4 Resource Optimization in Low-Income Settings

AI-driven PACS innovations, such as cloud-based accessibility and automated diagnostics, can significantly improve healthcare delivery in resource-limited settings. By reducing reliance on on-site expertise and infrastructure, these technologies enable equitable access to high-quality imaging services worldwide.

These broader implications underscore the transformative potential of AI integration, paving the way for a more interconnected, efficient, and patient-centered healthcare system.

##### 8.2.2 Recommendations for Stakeholders

To fully harness the potential of AI integration into PACS, collaborative efforts among stakeholders in healthcare are critical. The following specific actions are recommended:

Policymakers:

- Develop and implement standardized frameworks for AI validation and regulatory compliance, ensuring patient safety and data security.
- Promote funding initiatives to support research and development of AI technologies tailored to medical imaging.
- Establish guidelines for the ethical use of AI, focusing on data privacy, algorithmic fairness, and transparency.

Healthcare Institutions:

- Invest in IT infrastructure upgrades, including cloud computing and interoperability solutions, to support AI integration with PACS.
- Provide training programs for radiologists, technicians, and healthcare providers to improve their proficiency in AI-enabled tools.
- Establish multidisciplinary AI task forces to evaluate and oversee the implementation of AI solutions within the clinical workflow.

Industry Partners:

- Collaborate with healthcare providers to co-develop AI tools that address specific clinical challenges, ensuring relevance and usability.
- Prioritize the design of scalable, interoperable AI solutions that adhere to global standards such as DICOM and HL7.
- Provide post-deployment support, including software updates and performance monitoring, to ensure consistent functionality.

Educators and Academic Institutions:

- Integrate AI training into medical and radiology curricula, focusing on the practical applications and limitations of AI in clinical practice.
- Foster interdisciplinary collaborations between computer science and healthcare professionals to develop innovative AI solutions.
- Encourage academic research on the ethical, social, and economic implications of AI adoption in medical imaging.

Global Organizations and Nonprofits:

- Advocate for equitable access to AI technologies, particularly in low-resource settings, by facilitating knowledge sharing and partnerships.
- Support initiatives that develop cost-effective AI solutions tailored to underserved populations.

These actions collectively address technical, operational, and ethical barriers, paving the way for the successful integration of AI-powered PACS into healthcare systems worldwide.

##### 8.2.3 Developing Training Programs for Radiologists

The successful integration of AI tools into PACS systems requires radiologists to adapt to new workflows and acquire skills for leveraging AI effectively. Training programs tailored to this purpose are essential to ensure that radiologists remain central to the diagnostic process while maximizing the potential of AI-driven tools.

Key Objectives of the Training Programs:

- Enhance radiologists' understanding of AI algorithms, their capabilities, and limitations.
- Teach radiologists how to interpret AI-generated outputs, including confidence metrics and error analysis, to maintain clinical decision-making accuracy.
- Familiarize trainees with the ethical and regulatory implications of AI use, including data privacy, algorithmic bias, and accountability.
- Build confidence in utilizing AI-powered tools for tasks such as automated reporting, image segmentation, and predictive analytics.

Suggested Training Modules:

1. **Foundations of AI in Radiology**:

o Introduction to machine learning, deep learning, and their applications in PACS.

o Overview of common AI models and their use in medical imaging.

2. **Practical Applications and Hands-On Training**:

o Using AI-powered PACS for specific tasks, including automated workflows and diagnostic assistance.

o Case studies and simulation exercises to demonstrate real-world applications.

3. **Understanding Limitations and Bias**:

o Recognizing AI’s limitations in complex cases or rare conditions.

o Addressing algorithmic bias and ensuring diversity in data interpretation.

4. **Ethics and Accountability in AI**:

o Navigating regulatory compliance, data security, and patient confidentiality.

o Understanding the role of radiologists in overseeing AI-generated recommendations.

5. **Continuous Learning and Updates**:

o Periodic refreshers on advancements in AI technology and PACS systems.

o Interactive workshops and webinars with industry experts.

Proposed Implementation Strategies:

- Collaboration between radiology societies, academic institutions, and AI developers to design and standardize curricula.
- Incorporating these programs into radiology residency training and continuing medical education (CME) initiatives.
- Offering certifications through professional organizations, such as the Radiological Society of North America (RSNA), to ensure skill standardization.

These training programs will enable radiologists to effectively integrate AI tools into their workflows, ensuring improved diagnostic accuracy, patient outcomes, and overall efficiency in clinical practice.

## CONCLUSION

This narrative review synthesized current knowledge on AI integration into PACS, marking a transformative leap in medical imaging and enhancing diagnostic capabilities, workflow efficiency, and patient care. AI technologies such as deep learning, natural language processing, and federated learning have advanced image analysis, automated reporting, and data privacy, addressing longstanding challenges in radiology. However, regulatory compliance, data standardization, and interoperability remain critical barriers to effective adoption.

Ongoing innovation and collaboration among clinicians, researchers, and industry stakeholders are essential to harness the full potential of AI-enhanced PACS, paving the way for more accurate, efficient, and personalized diagnostic imaging in healthcare.

## DECLARATION OF GENERATIVE AI AND AI-ASSISTED TECHNOLOGIES

During the preparation of this manuscript, the authors used the Grammarly software to assist with grammar and formatting. The authors reviewed and edited the content following the software's suggestions and took full responsibility for the final version of the manuscript.

## Figures and Tables

**Fig. (1):**
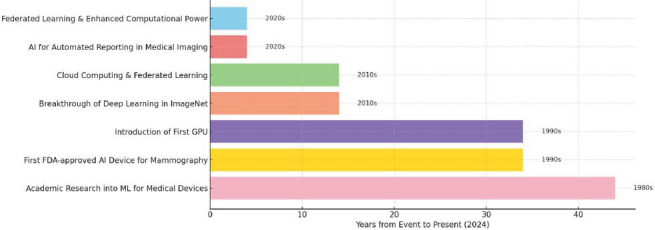
Timeline of Key AI and ML Milestones in Medical Imaging. This figure presents a timeline of significant milestones in the integration of artificial intelligence (AI) and machine learning (ML) into medical imaging, ranging from early research in the 1980s to recent advances in federated learning and enhanced computational power in the 2020s. Each milestone reflects the transformative steps that have shaped AI-powered diagnostic capabilities, enabling more accurate and efficient workflows within PACS.

**Fig. (2):**
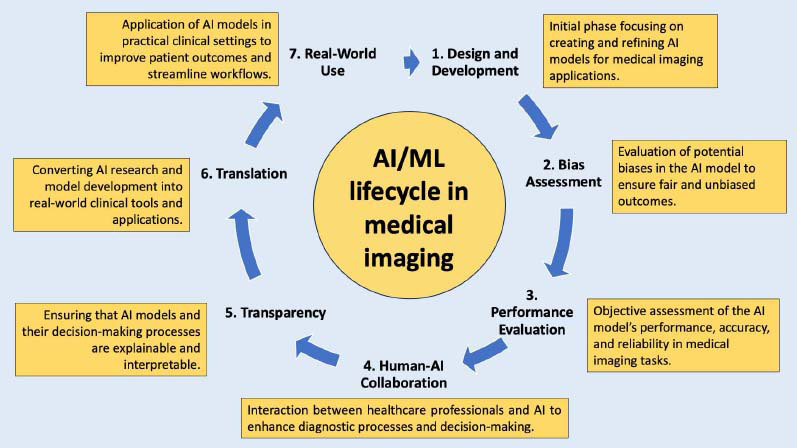
AI/ML Lifecycle in Medical Imaging. Key Stages from Development to Clinical Integration. This figure illustrates the lifecycle of artificial intelligence (AI) and machine learning (ML) applications in medical imaging, highlighting seven essential stages: (1) Design and Development, focusing on the creation and refinement of AI models; (2) Bias Assessment, evaluating potential biases to ensure fairness; (3) Performance Evaluation, objectively measuring accuracy and reliability; (4) Human-AI Collaboration, enhancing diagnostic processes through interaction between clinicians and AI tools; (5) Transparency, ensuring explainable and interpretable decision-making; (6) Translation, converting AI research into practical clinical applications; and (7) Real-World Use, applying AI models in clinical settings to improve patient care and streamline workflows. The circular arrangement signifies the continuous process of refinement and integration of AI technologies in medical imaging.

**Fig. (3):**
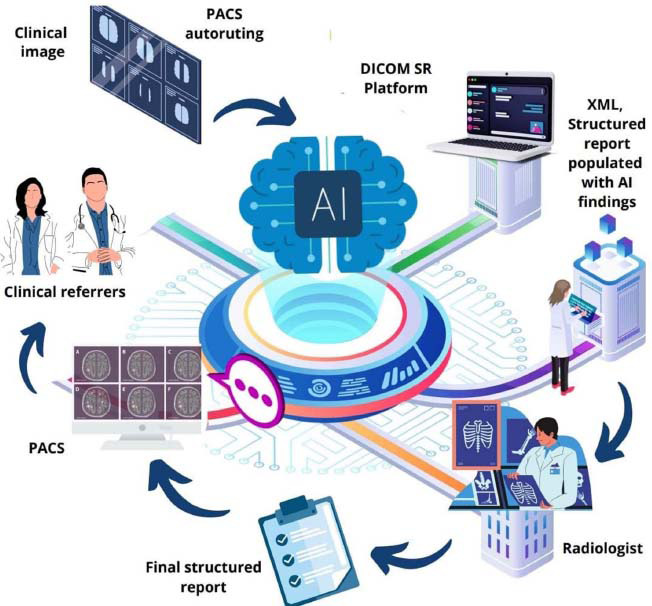
Workflow of AI-Powered Structured Reporting in PACS. This illustration depicts the end-to-end workflow of integrating AI into PACS, from initial image acquisition to final structured reporting. Key stages include AI-assisted image analysis, data processing within a DICOM SR platform, and XML-based structured reporting populated with AI findings, enabling seamless communication between radiologists, clinical referrers, and PACS systems for enhanced diagnostic accuracy and efficiency. AI: Artificial Intelligence; PACS: Picture Archiving and Communication Systems; DICOM: Digital Imaging and Communications in Medicine; SR: Structured Reporting; XML: Extensible Markup Language.

**Table 1:** Evolution of PACS Over Five Decades: Key Technological Milestones and AI Integration. This table summarizes the significant technological milestones in PACS development and the integration of AI from the 1970s to the present, highlighting the impact on clinical practice, diagnostic capabilities, and workflow efficiency.

**Decade**	**Technological Milestones**	**Key Developments in AI Integration**	**Clinical Impacts**
1970s	- Early development of PACS (Picture Archiving and Communication Systems) concept.	- AI was largely absent in healthcare and PACS development during this era.	- Introduction of digital image storage and communication, replacing analog film-based systems (2000 PACS - filmless radiology, 2000).
1980s	- Adoption of DICOM (Digital Imaging and Communications in Medicine) standard.	- Basic image processing algorithms used for image enhancement and compression.	- Improved interoperability between imaging modalities (MRI, CT, X-ray).
	- Improved image compression techniques and digital image archiving.	- First computer-aided detection systems explored for limited applications.	- Facilitated the storage, retrieval, and sharing of digital images across institutions.
1990s	- Wide adoption of PACS across hospitals for medical imaging.	- Introduction of early machine learning algorithms for image analysis.	- Enhanced diagnostic capabilities with early automated detection tools.
	- Integration with Radiology Information Systems (RIS) and Hospital Information Systems (HIS).	- Emergence of computer-aided diagnosis (CAD) for mammography and lung nodule detection.	- Increased efficiency in imaging workflows (2000 PACS - filmless radiology, 2000).
2000s	- Advances in PACS storage capacity with the shift to cloud-based PACS.	- Computer vision techniques applied for segmentation and pattern recognition.	- Enhanced remote access and sharing of medical images.
	- Use of web-based PACS viewers to enable remote access to images.	- Introduction of deep learning techniques in research for radiology image analysis.	- Workflow optimization through cloud-based storage (2019 Integrating a Cloud-Based PACS Viewer, 2019).
	- Rise of digital radiography (DR).	- Gradual AI integration for workflow improvements.	
2010s	- PACS systems fully integrated with electronic health records (EHR) and RIS.	- Rapid development of deep learning and convolutional neural networks (CNNs) for medical image analysis.	- Improved diagnostic accuracy and efficiency.
	- Advancements in imaging modalities: MRI, CT, and PET.	- AI was applied in the segmentation, classification, and detection of lesions in medical images.	- Automated reporting systems and enhanced image analysis capabilities.
	- Growth in teleradiology.	- AI-powered PACS systems developed to assist in image triage and prioritization (2022 State-of-the-Art AI in MRI, 2022).	- Expansion of AI applications in radiology.
2020s (Present)	- Increased reliance on cloud-based PACS and edge computing for faster processing.	- Artificial intelligence now assists in nearly all aspects of image management and diagnosis (*e.g.*, triage, segmentation, classification).	- Significant reduction in diagnostic errors and improved radiologist productivity.
	- AI fully integrated into PACS for workflow optimization.	- AI enables real-time analysis of medical images and predictive analytics.	- AI-driven personalized treatment planning and predictive analytics in clinical care.
	- Greater interoperability.	- Natural language processing (NLP) is used for automated report generation (PACS-AI Platform, 2024).	

**Table 2:** AI-Powered PACS Applications Across Different Imaging Modalities. Overview of various AI techniques used in PACS, categorized by application goals, imaging modalities, network architectures, and additional data sources, emphasizing advancements in automated analysis, segmentation, and clinical support.

**Reference**	**Goal**	**Technique**	**Images Modality**	**Network Architecture**	**Additional**
Beetz, 2022 [185]	Analysis of Body Composition	Automatic Segmentation	200 CT (L3) slides	CNN: U-Net	Metabolic data
Yepes, 2018 [38]	Measure brain ventricular volume	Decrypt and Uncompress	MRI	Support Vector Machine (SVM)	-
Yang, 2020 [186]	Extract multi-source data for diagnosis	AI analysis & Info extractor	Digital X-ray/MRI/CT/US	Natural Language Processing (NLP)	HIS-RIS data
Navab NJH, 2015 [187]	Delimit Neural Structures	Segmentation	Electron Microscopic	CNN: U-Net	-
Zhang, 2020 [39]	Delimit Liver Tumor	Segmentation	CT (LiTS2017 & 3D-IRCADb 2019 databases)	CNN: V-Net based on distance metric	-
Sridhar, 2022 [188]	Reduce storage, processing, and transmission	Compression	DR/CT/Mammograms	Recurrent-Net GenPSOWVQ (with wavelet)	-
Chen, 2021 [189]	Testing for COVID-19	Classification	CT	CNN-Ensemble (VGG-19, Resnet-101, DenseNet-201, Inception-v3) (voting strategy)	-
Halabi, 2019 [190]	Bone age assessment	Prediction	DR-Hand	Inception-v3 & DenseNet-32	-
Arunachalam, 2019 [191]	Identify viable and necrotic areas on osteosarcoma	Detection & Classification	Histology slides	SVM & CNN	-
Sayres, 2019 [192]	Grading for Diabetic Retinopathy	Classification	Retina images	CNN	-
Pinto, 2019 [193]	Structure report	Info extractor	DR	CNN	-
Kovacs, 2017 [194]	Correlate PACS-HIS to clinical follow-up	Info extractor	-	Natural Language Processing (NLP)	Electronic Medical Records (EMR)

**Table 3:** Recommended security measures for PACS data protection include data encryption, access control, risk management, and compliance with regulatory standards such as DICOM, HIPAA, and ISO 27000.

**Recommendation**	**Action**	**Example**
Establish security policies	Risk Management	Medical device asset management
	Perform backup, redundancy, and automatic recovery	Controlling secure sensitive data in third-party cloud storage
Application firewalls, proxy servers, and network inspection tools	Components Controls	Control of third-party practices or downstream suppliers who may implement malicious technology
Data Encryption	Remote backup of encrypted copies	
Communications Capabilities	Interoperability with clinical systems like RIS	Control of network traffic: network zoning, denial of service when network infrastructure is saturated, or IP addresses can be disrupted
Establish Access Control Mechanisms	Authentication Mechanisms limiting remote technical support	For access to imaging devices and clinical systems
Define User Roles with different privileges	Role definition for radiologists, technicians, clinicians, and administrators	Radiologist: can write radiology reports; Technician: can confirm the patient exam but cannot write reports; Clinician: can view reports and images; Administrator: can configure and manage the entire PACS
Interoperability	Intranet PACS support for role-based access controls	PACS-RIS-HIS intranet
	Internet PACS support for secure communication, data protection, and access controls	PACS Web Internet: cloud storage capabilities, SSL-based communication channels
Meet Standards	Interface that allows clinical systems (HIS and RIS) to interact with PACS	DICOM, HL7, ISO27000, HIPAA

**Table 4:** AI Innovations in MRI Applications Across Medical Specialties. Summary of AI methods utilized in MRI across medical specialties, detailing clinical applications, disease focus, and relevance to diagnostic accuracy and patient management.

**Speciality**	**AI Method**	**Clinical Disease**	**Relevance**
**Breast Imaging**	Deep Learning, Machine Learning	Breast cancer detection	Improves breast cancer detection accuracy, risk assessment, and decision support [100].
**Pelvic MRI**	Convolutional Neural Networks (CNN), Deep Learning Reconstruction (DLR)	Prostate cancer, Bladder, Uterus, Ovaries, Rectum disorders	Enhances lesion detection, organ segmentation, and risk stratification in pelvic organs [101].
**Neuroradiology**	Deep Neural Networks (DNN), Artificial Neural Networks (ANN)	Multiple Sclerosis (MS), NMOSD, Brain lesions	Ethical challenges and advanced anomaly detection in brain imaging [103].
**Brain Tumors**	Machine Learning (ML), Radiomics, Deep Learning (DL)	Gliomas (*e.g.*, Glioblastoma)	Predicts survival and stratifies patients into survival groups [104].
**Prostate MRI**	Computer-Aided Detection (CAD), Deep Learning (DL)	Prostate cancer detection and quality control	Reduces variability and improves diagnostic accuracy of prostate MRI [105].
**Radiotherapy (MRI-guided)**	Motion tracking and prediction via AI (Deep Learning, Machine Learning)	Tumor motion management in radiotherapy	Improves real-time motion management and treatment accuracy during radiotherapy [107].
**Cardiac MRI**	Deep Learning (DL), Machine Learning (ML)	Cardiovascular diseases	Enhances diagnostic precision and reduces acquisition time in cardiovascular disease management [108].

## LIST OF ABBREVIATIONS

AIMD: Artificial Intelligence Medical Devices
ANN: Artificial Neural Network
AR: Augmented Reality
BERT: Bidirectional Encoder Representations from Transformers
CAD: Computer-Aided Diagnosis
CNN: Convolutional Neural Network
CT: Computed Tomography
DICOM: Digital Imaging and Communications in Medicine
DL: Deep Learning
DR: Digital Radiography
DXA: Dual-Energy X-ray Absorptiometry
EHR: Electronic Health Record
FDA: Food and Drug Administration
FHIR: Fast Healthcare Interoperability Resources
GAN: Generative Adversarial Network
GPU: Graphics Processing Unit
HIS: Hospital Information System
HL7: Health Level Seven
ICH: Intracranial Hemorrhage
ISO: International Organization for Standardization
LAN: Local Area Network
ML: Machine Learning
MRI: Magnetic Resonance Imaging
NLP: Natural Language Processing
PACS: Picture Archiving and Communication Systems
PE: Pulmonary Embolism
PET: Positron Emission Tomography
PCC: Patient-Centered Care
RIS: Radiology Information System
SNA: Radiological Society of North America
SR: Structured Reporting
SVM: Support Vector Machine
VR: Virtual Reality
WAN: Wide Area Network
XAI: Explainable Artificial Intelligence
